# Supplementary figures and images for: GM-CSF and M-CSF Driven Differentiation Differentially Regulates Chikungunya Virus Infection and Antiviral Responses in Human Monocyte-Derived Macrophages
Source: bioRxiv. 2026 Mar 12:2026.03.11.710213. Preprint. [Version 1] doi: 10.64898/2026.03.11.710213 (PMC13060824; doi:10.64898/2026.03.11.710213)

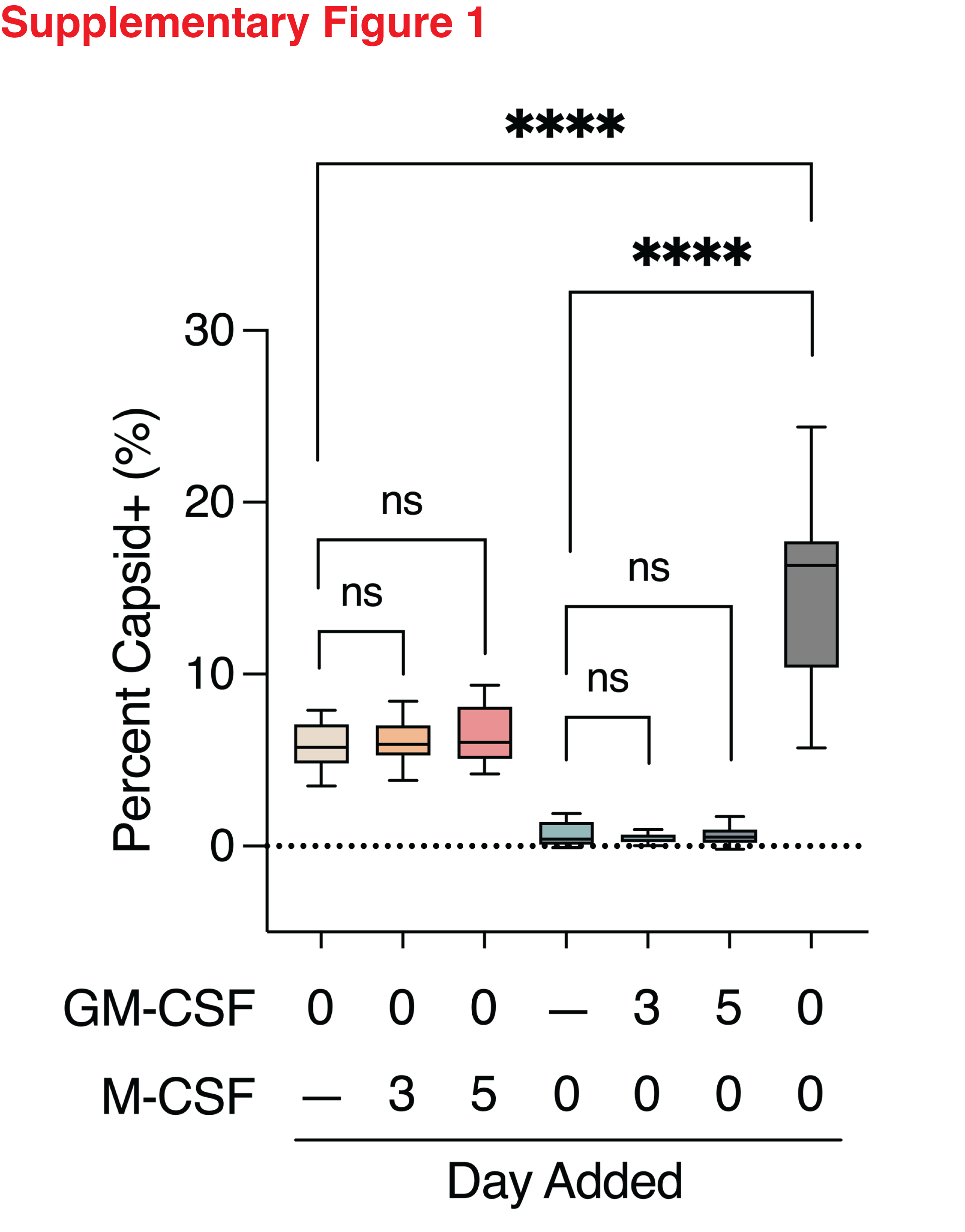

Supplement: Supplement 1 — S1 Fig. Temporal addition of GM-CSF or M-CSF on CHIKV infectivity in primary macrophages. Monocyte-derived macrophages were differentiated with GM-CSF, M-CSF, or both cytokines and infected with CHIKV 181/25 (MO1=1.0) for 24 hours. To assess whether susceptibility to CHIKV can change, macrophages were treated with the opposite cytokine at 0-, 3-, or 5-days post differentiation. Quantification of capsid positive macrophages was detected via immunofluorescence. Percent infection was determined via the quotient of number of capsid positive cells divided by the number of DAPI positive cells. Data represented as means ± SEM (n= 2 donors). Statistical analysis was performed via one-way ANOVA with Fisher’s LSD test. Statistical analysis was performed via one-way ANOVA with Geisser-Greenhouse correction and Fisher’s LSD test. Black asterisks represent P-value statistical significance, where ns=non-significant, *=P<0.05, **=P<0.01, ***=P<0.001, and ****=P<0.0001. [file media-1.tif]

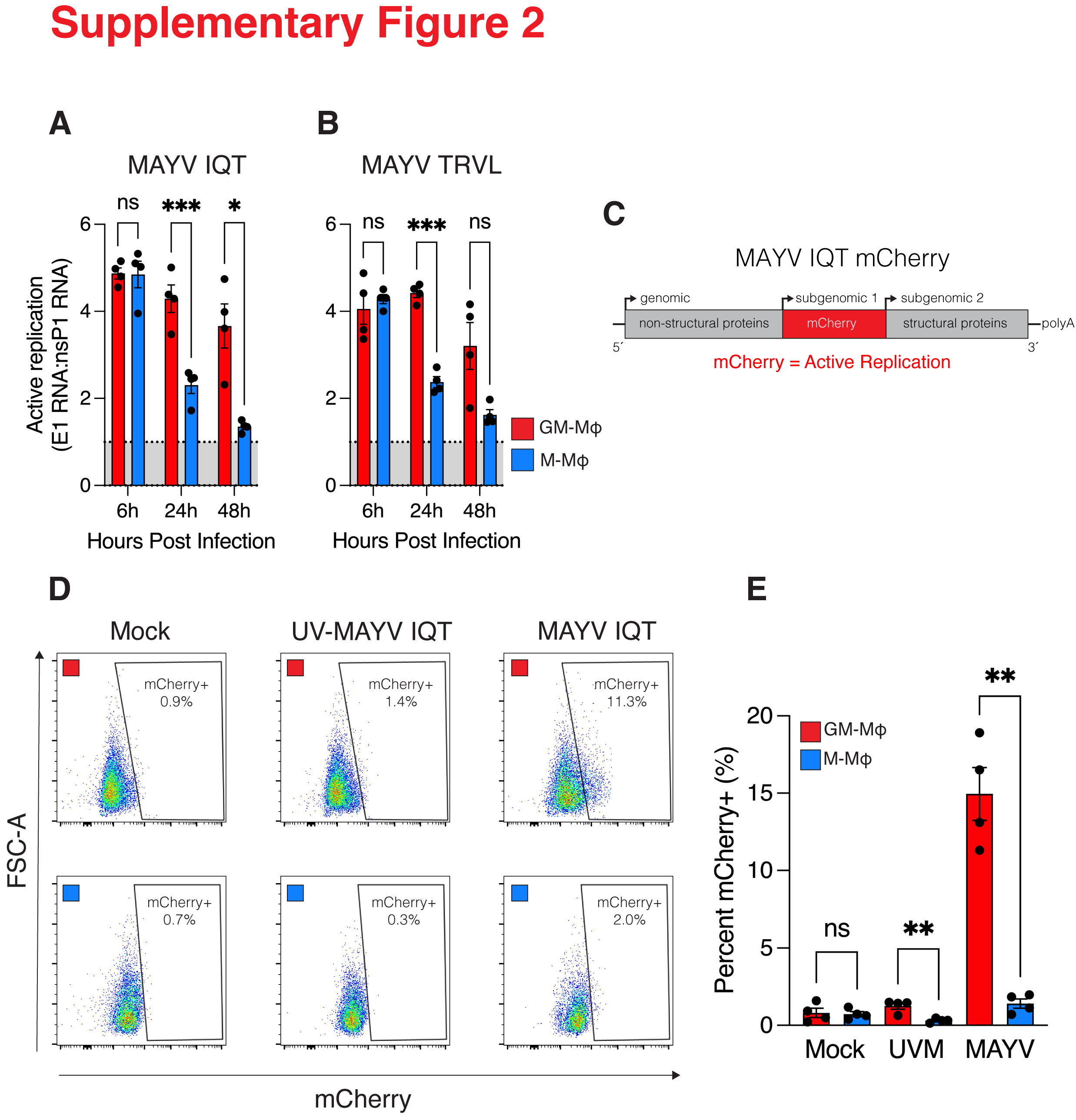

Supplement: Supplement 2 — S2 Fig. Viral infectivity and replication of Mayaro virus in GM-Mϕ and M-Mϕ. (A-B) GM-Mϕ (red bars) or M-Mϕ (blue bars) for 6 days and infected with MAYV TRVL or MAYV IQT (MOI=1.0) for 6, 24, and 48 hours. Intracellular viral replication is represented as “active replication”, which was determined by evaluating the ratio of E1 subgenomic viral RNA copies to nsP1 genomic viral RNA copies. Concentration of viral RNA was determined by RT-qPCR via standard curve. Statistical analysis was performed via two-way ANOVA with Geisser-Greenhouse correction and Fisher’s LSD test. Data represented as means ± SEM (n=4 donors). (C) Schematic representation of infectious clone encoding MAYV-IQT-mCherry reporter virus, where the mCherry protein is expressed under the control of a subgenomic promoter. (D) GM-Mϕ (top row) and M-Mϕ (bottom row) were infected with mock, UV-inactivated MAYV IQT-mcherry (MOI=1.0), or MAYV IQT-mCherry (MOI=1.0) for 24 hours and prepared for flow staining. Flow plots represent mCherry+/LIVE-DEAD-/Singlet. Flow plots are representative of one donor. (E) Quantification of mCherry+ mock, UV-inactivated MAYV IQT-mCherry, or MAYV IQT-mCherry GM-Mϕ and M-Mϕ determined via flow cytometry. Data represented as means ± SEM (n=4 donors). Statistical analysis was performed via one-way ANOVA Geisser-Greenhouse correction and Fisher’s LSD test. Black asterisks represent P-values, where ns=non-significant, *=P<0.05, and **=P<,0.01, ***=P<0.001, ****=P<0.0001. [file media-2.tif]

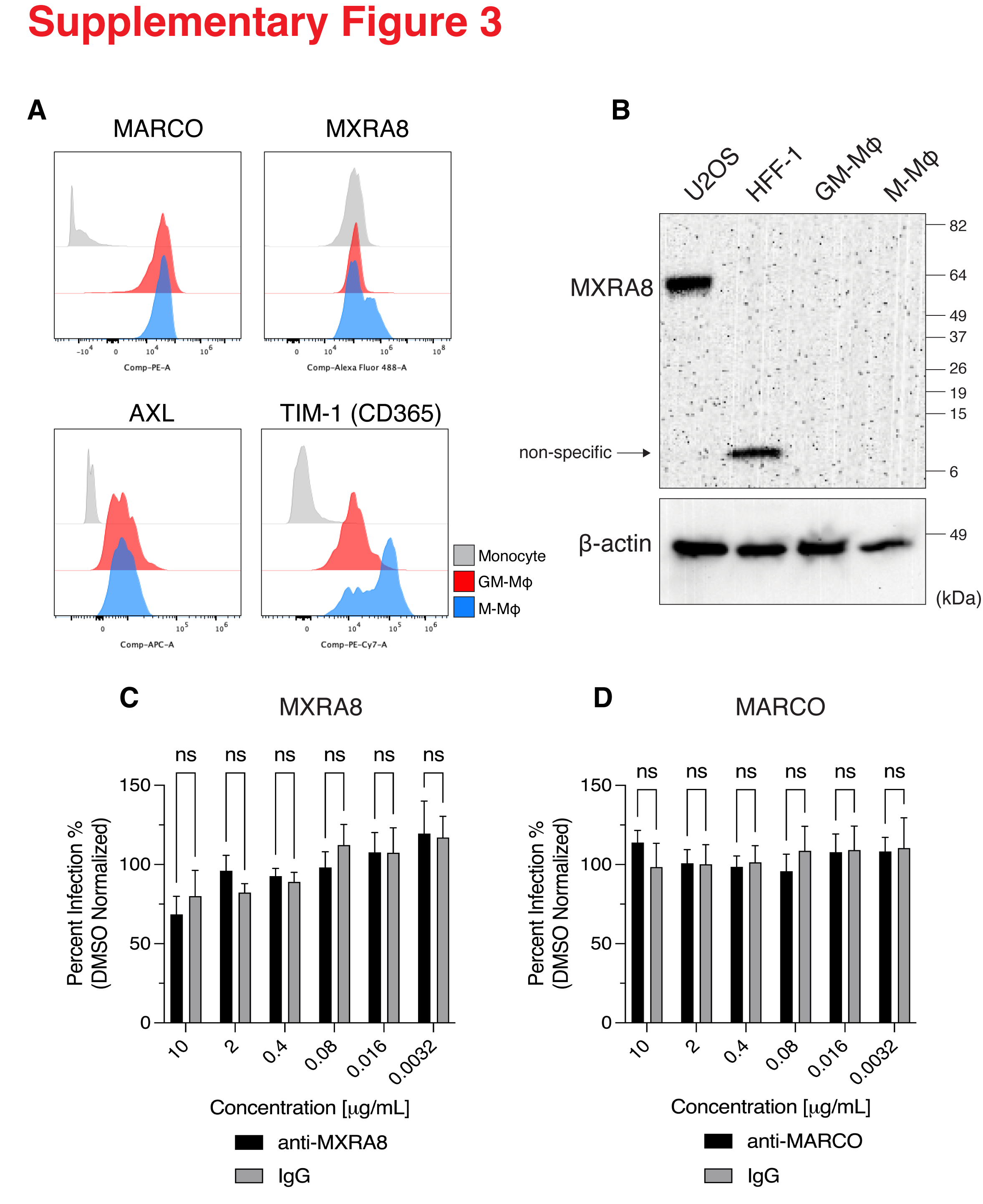

Supplement: Supplement 3 — S3 Fig. Evaluation of alphavirus receptor expression in GM-Mϕ and M-Mϕ. GM-Mϕ (red), M-Mϕ (blue), and undifferentiated monocytes (gray) were surface stained with a panel of antibodies assessing the expression of alphavirus receptors (A) MARCO, MXRA8, AXL, and TIM-1 (CD365). Histograms are representative of a single donor. (B) Immunoblot analysis of total MXRA8 and β-Actin protein expression in un-infected U2OS, HFF-1, GM-Mϕ and M-Mϕ. Data is representative a single replicate (cell lines) and single donor (macrophages). GM-Mϕ were pre-treated with media containing mAbs (up to 10 μg/mL) against (C) MXRA8 or (D) MARCO and respective IgG isotype controls. Cells were then infected with CHIKV 181/25 (MOI=2.0) for 1 hour, followed by re-supplementation with media containing antibodies for a total incubation time of 24 hours. To determine percent infection, immunofluorescence staining of CHIKV capsid protein to determine the number of infected cells and DAPI to determine total cell count. Infection data from antibody treatment is represented as relative percent infection normalized to DMSO control treatment (n=4 donors). Statistical analysis was performed via two-way ANOVA Geisser-Greenhouse correction and Fisher’s LSD test. Black asterisks represent P-values, where ns=non-significant. [file media-3.tif]

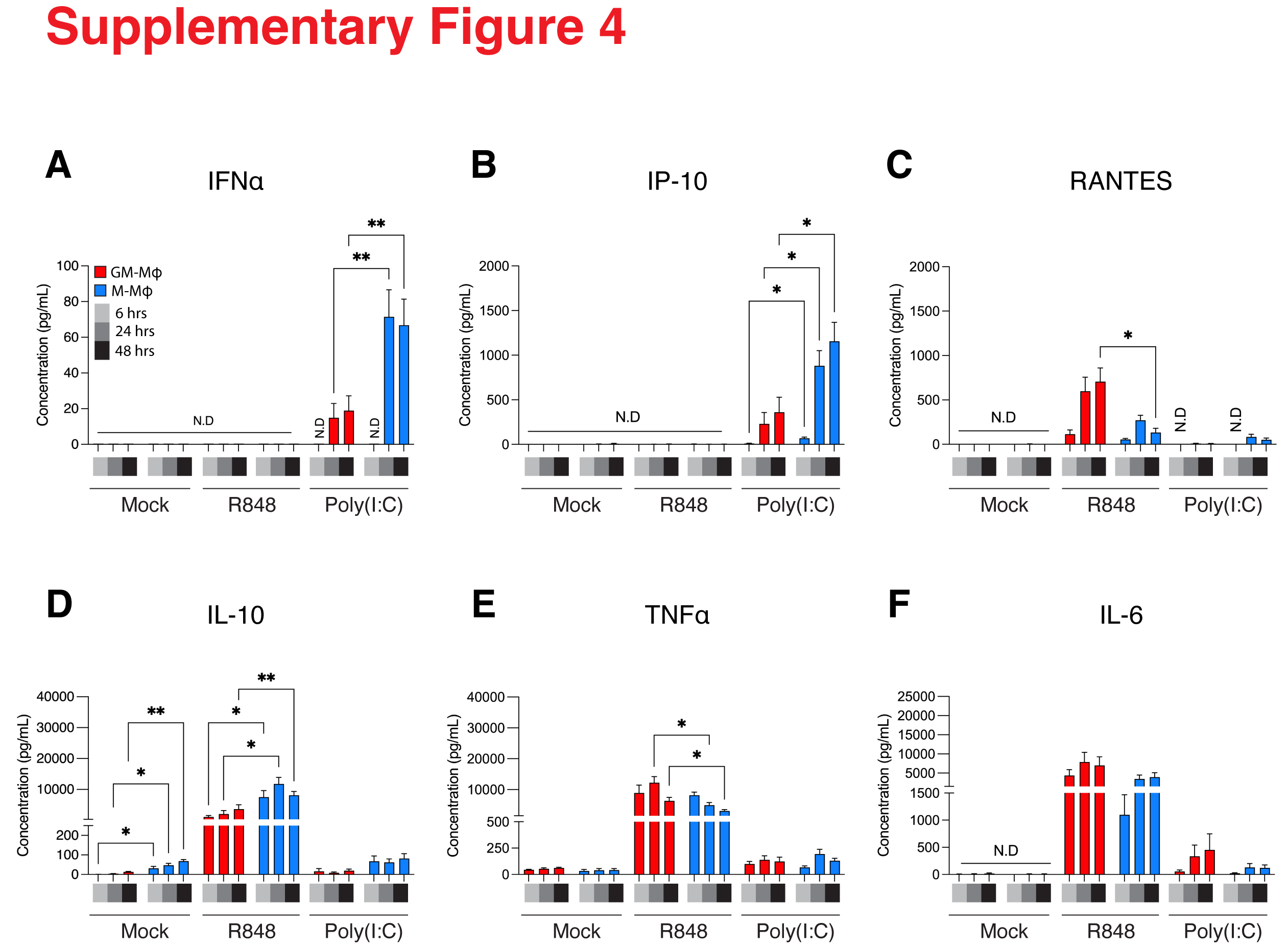

Supplement: Supplement 4 — S4 Fig. Cytokine and chemokine secretion in supernatant of GM-Mϕ and M-Mϕ following R848 or poly(I:C) stimulation. GM-Mϕ (red bars) or M-Mϕ (blue bars) for 6 days and treated with 1 μg/mL of R848 or poly(I:C) for 6, 24, and 48 hours. Multiplex ELISA was performed to evaluate secretion of select cytokines and chemokines: (A) IFNα, (B) IP-10/CXCL10, (C) RANTES/CCL5, (D) IL-10, (E) TNFα, and (F) IL-6. Non-detected (N.D.) indicates values below limit of detection as determined via Belysa analysis software. Data represented as means ± SEM where n=4 donors. Statistical analysis was performed via two-way ANOVA with Geisser-Greenhouse correction and Fisher’s LSD test. Black asterisks represent P-value statistical significance, where *=P<0.05, and **=P<0.01. Statistical comparisons resulting in non-significant P-values (>0.05) were performed but not reported. [file media-4.tif]
